# Update of the evidence- and consensus-based S3 guideline on atopic dermatitis: Systemic therapy with biologics or Janus kinase inhibitors and specific aspects of systemic therapy in pregnancy and lactation 

**DOI:** 10.5414/ALX02638E

**Published:** 2026-06-15

**Authors:** Thomas Werfel, Annice Heratizadeh, Matthias Augustin, Christine Bangert, Andrea Bauer, Tilo Biedermann, Richard Brans, Nadine Domröse, Uwe Gieler, Oliver Gießler-Fichtner, Eckard Hamelmann, Selina Hampe, Ruben Heuer, Julia Kahle, Maria Kinberger, Markus Koch, Meike Köhler, Franz Legat, Katja Nemat, Irena Neustädter, Eva M. J. Peters, Susanne Radonjic-Hoesli, Imke Reese, Peter Schmid-Grendelmeier, Uta-Katharina Schmidt-Göhrich, Jochen Schmitt, Christina Schnopp, Thomas Schwennesen, Dagmar Simon, Kristina Stamos, Christian Termeer, Regina Treudler, Ralph von Kiedrowski, Iris Wagner, Anja Waßmann-Otto, Gesine Weckmann, Ricardo Niklas Werner, Andreas Wollenberg, Margitta Worm, Hagen Ott

**Affiliations:** 1 Department of Dermatology and Allergy, Hannover Medical School, Hannover,; 2 Competence Center for Health Services Research in Dermatology (CVderm), Institute for Health Services Research in Dermatology and Nursing (IVDP), University Medical Center Hamburg-Eppendorf, Hamburg, Germany,; 3 Department of Dermatology, Medical University of Vienna, Vienna, Austria,; 4 Department of Dermatology, Faculty of Medicine and University Hospital Carl Gustav Carus, Technische Universität Dresden, Dresden,; 5 TUM University Hospital, Department of Dermatology and Allergy, Munich,; 6 Institute for Interdisciplinary Dermatological Prevention and Rehabilitation (iDerm) at the Osnabrück University, Osnabrück,; 7 Department of Psychosomatic Medicine and Psychotherapy, University Hospital Gießen, Gießen,; 8 Gaißach Specialist Clinic of DRV Bayern Süd, Gaißach,; 9 Children’s Center, Evangelical Hospital Bethel, University Hospital OWL, University of Bielefeld, Bielefeld,; 10 German Allergy and Asthma Association (DAAB), Mönchengladbach,; 11 Department of Dermatology, Venereology and Allergology, Division of Evidence Based Medicine in Dermatology (dEBM), Charité - Universitätsmedizin Berlin, corporate member of Freie Universität Berlin and Humboldt-Universität zu Berlin, Berlin,; 12 Alpenklinik Santa Maria, Bad Hindelang,; 13 Section for Integrated Pediatric Dermatology (iKinD), Munich Center for Children with Medical and Developmental Complexity, LMU University Hospital, Munich, Germany,; 14 Department of Dermatology and Venereology, Medical University of Graz, Graz, Austria,; 15 Practice for pediatric pneumology and allergology, Children’s Center Dresden-Friedrichstadt (Kid),; 16 Department of Pediatrics, Faculty of Medicine and University Hospital Carl Gustav Carus, Technische Universität Dresden, Dresden,; 17 Hospital Hallerwiese, Cnopfsche Kinderklinik, Nuremberg,; 18 Psychoneuroimmunology Laboratory, Department of Psychosomatic Medicine and Psychotherapy, Justus-Liebig University Gießen, Gießen, Germany,; 19 Department of Dermatology, Inselspital Bern, Bern, Switzerland,; 20 Private Practice for Dietary Advice and Nutrition Therapy with Special Interest in Adverse Reactions to Food, Munich, Germany,; 21 Allergy Unit, Department of Dermatology, University Hospital Zurich and Christine Kuehne Center for Allergy Research and Education CK-CARE Davos, Switzerland,; 22 Carus Family Practice at Dresden University Hospital,; 23 Center for Evidence-Based Healthcare (ZEGV), University Hospital Dresden and Medical Faculty Carl Gustav Carus, Technical University Dresden, Dresden,; 24 German Eczema Association (DNB), Hamburg,; 25 Dermatology Practice at Löwenmarkt, Stuttgart-Weilimdorf,; 26 Department of Dermatology, University-Hospital Freiburg, Freiburg,; 27 Institute of Allergology, Charité – Universitätsmedizin Berlin, Corporate Member of Freie Universität Berlin and Humboldt-Universität zu Berlin, Berlin,; 28 Selters Dermatology Practice, Selters,; 29 Mensing Derma MVZ, Hamburg,; 30 Weckmann Institute of Medical and Healthcare Education, Rostock,; 31 Department of Dermatology and Allergology, University Hospital Augsburg, Augsburg,; 32 Clinic and Polyclinic for Dermatology and Allergology, Ludwig Maximilian University, Munich,; 33 Department of Dermatology, Venereology, and Allergology, Charité – Universitätsmedizin Berlin, corporate member of Freie Universität Berlin and Humboldt-Universität zu Berlin, Berlin, and; 34 Department of Pediatric Surgery, Dr. Von Hauner Children’s Hospital, LMU University Hospital, Munich, Germany; * Thomas Werfel, Annice Heratizadeh and Hagen Ott have contributed equally as a coordinating team to this guideline.

## Abstract

This guideline is a partial update of the S3 guideline on atopic dermatitis (AWMF register no. 013-027) published in 2023. The chapters on systemic therapy with biologics and Janus kinase inhibitors as well as the chapter on pregnancy, breastfeeding and family planning in the context of systemic therapies for atopic dermatitis have been updated. This was prompted by new approvals (lebrikizumab, nemolizumab), approval extensions (abrocitinib from 12 years of age, baricitinib from 2 years of age) and new evidence on the use of biologics before and during pregnancy. In addition, a new chapter on treatment goals, treatment expectations and criteria for treatment adjustment (“treat-to-target”) in systemic therapies has been added to the guideline. This article only presents the updated and newly added chapters. The complete guideline is available on the AWMF website.[Table Table4]

AWMF-Register Nr.: / AWMF Registry No.: 013-027

## 1. Introduction 

This guideline represents an update of the S3 guideline on atopic dermatitis (AD) published in 2023. It focuses on systemic therapies with biologics and Janus kinase (JAK) inhibitors, as well as systemic therapies for pregnant and breastfeeding individuals with AD [1, 2]. Systemic therapies with azathioprine, cyclosporine, methotrexate and mycophenolate mofetil (conventional systemic therapies) are listed only in Table 1 and Table 2 and are addressed in the sections on pregnancy and breastfeeding. They are not discussed in detail here, as there have been no significant changes since the 2023 guideline [1, 2]. 

Some sections of the text were taken from previous versions without any changes to improve understanding of the methodology of the guideline. In this work, the background texts of the chapters are significantly shorter and more concise than in the long version of this update. The long version contains information on dosages, therapy combinations, and further background information on systemic therapies and non-updated chapters on other aspects of AD management. The chapters and recommendations revised by the coordinating team were voted on in a two-stage online process via LimeSurvey. All experts nominated for the update reviewed the changes to the background texts, as well as the chapter recommendations and could vote to approve, reject, or abstain. If an expert rejected or abstained, an alternative proposal was requested and voted on in the second round. All nominated experts were eligible to vote. For final approval, only the votes of experts with no or only low conflicts of interest were taken into account, except for the chapter “Therapeutic Goals, Treatment Expectations, and Adjustment Criteria (“treat-to-target”) in Systemic Therapies for AD” on which all delegates were able to vote. During the voting process, experts could not see how the other experts had voted. If the recommendations are evidence-based according to the evidence report for this guideline, this is explicitly noted (https://register.awmf.org/de/leitlinien/detail/013-027). The updated evidence report presents the results of the network meta-analyses by Drucker et al. (https://eczematherapies.com/) [3, 4]. These analyze the effectiveness of biologics and JAK inhibitors compared to placebo and to each other based on controlled clinical studies within the first four months of treatment. The endpoints considered were eczema severity, pruritus and quality of life. 

After internal and external review, the updated recommendations and guideline texts were approved by the boards of all participating professional societies. The German Society for General Medicine and Family Medicine (DEGAM) abstained from voting on all evidence-based recommendations for biologics or JAK inhibitors, which did not affect the calculation of the degree of consensus. 

In this guideline, as in the previous version, strength of recommendations were standardized according to the AWMF recommendation. 

## 2. Indication for systemic therapy in AD, therapy goals, therapy expectations, and adjustment criteria (“treat-to-target”) for systemic therapy of AD[Table Table9]

Systemic therapy for AD is indicated when the disease cannot be adequately controlled with topical treatments – or, in adults, with UV light therapy. Systemic therapy can also be useful to reduce the overall amount of topical corticosteroids (TCS) in AD patients who require large amounts of potent TCS over extended periods. 

Systemic treatment is generally indicated for patients with a high total score (scale definition), patients who do not respond to properly administered topical or UV therapy (functional definition), or patients who are unable to participate in daily activities despite following an appropriate treatment regimen (social definition). 

**Figure 1.**
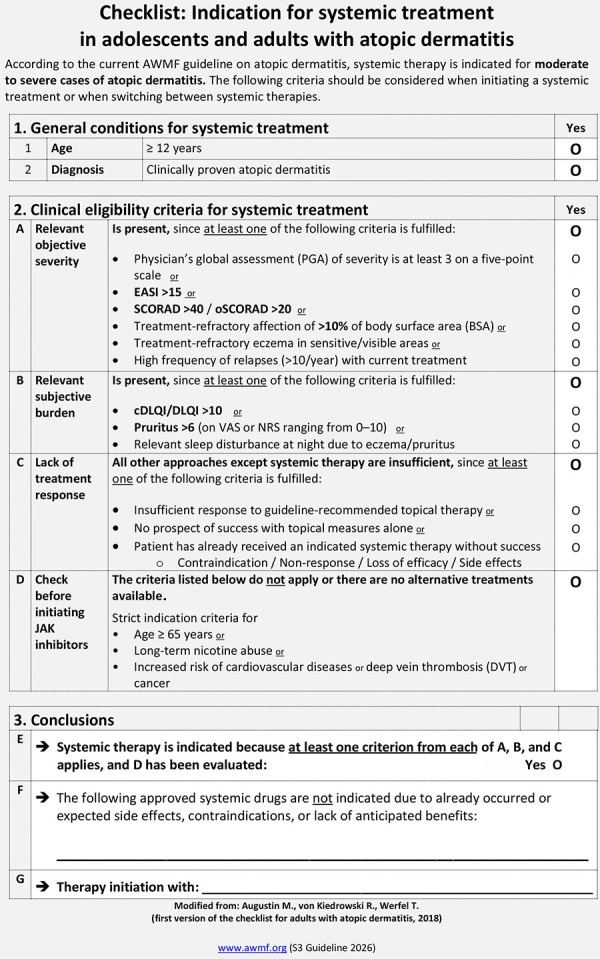
Checklist: Indication for systemic treatment of atopic dermatitis in adolescents and adults.

**Figure 2.**
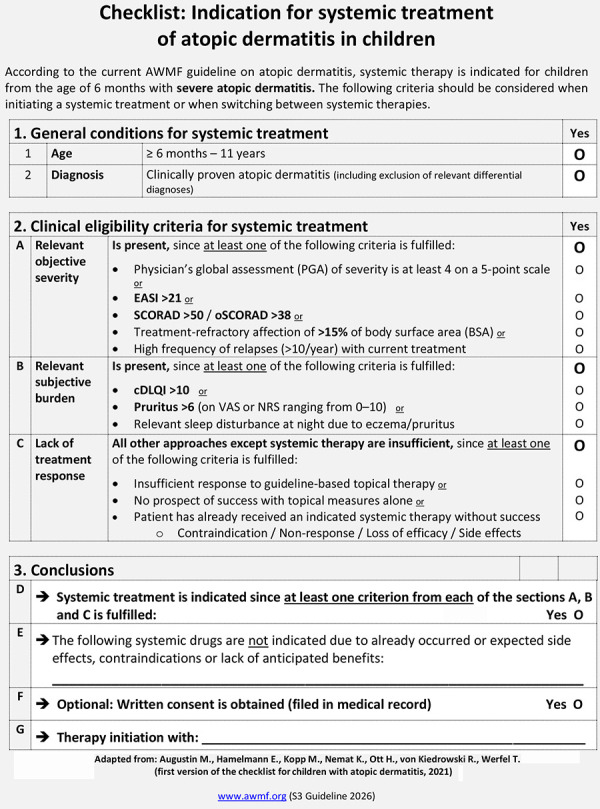
Checklist: Indication for systemic treatment of atopic dermatitis in children.

The indication for systemic therapy and the patient’s response to topical and systemic treatments should be recorded and documented in a suitable manner in clinical practice. Standardized documentation of the indication for systemic therapy in AD should be performed. Checklists for the indication and documentation of systemic therapy for AD in the age groups of children up to 11 years of age, as well as adolescents aged 12 years and older, and adults are shown in Figures 1 and 2. To fulfill the indication for systemic therapy for AD, at least one of the criteria for objective severity, subjective burden, and lack of treatment response must be met according to the checklist. 

The primary goal of the therapy of AD is to eliminate all signs and symptoms of the disease and the associated impairment of quality of life. In clinical practice, however, this ideal goal can often not fully be achieved even with modern systemic therapies combined with topical anti-inflammatory agents and emollients. It is therefore important to set realistic goals over time and to define objective criteria that will trigger adjustments to the treatment approach. To promote adherence and self-efficacy in the context of informed decision-making, treatment goals and expectations – as well as the course of action in the event they are not met – should be discussed and established together with the patients. 

The consented recommendations in this chapter of the guideline are based on the recommendations that have been made in Europe, as part of a consensus conference based on a Delphi method [5].

## 3. Overview of recommendations for long-term systemic anti-inflammatory therapy 

Table 1 provides an overview of recommendations for long-term anti-inflammatory systemic therapy in adult patients, and Table 2 provides recommendations for children and adolescents, as well as pregnant and breastfeeding patients with AD.

## 4. Interval or long-term therapy with biologics 

### 4.1. Dupilumab[Table Table3]

**Mechanisms of action and efficacy**

Dupilumab is a fully human IgG4 monoclonal antibody (mAb) approved for the treatment of AD. It has been available in Germany since the end of 2017 for the treatment of adults and is now also approved for children and adolescents aged six months and older. 

Dupilumab binds to the α subunit of the IL-4 receptor, which is part of both the IL-4 and IL-13 receptor complexes. The safety and efficacy of dupilumab were primarily demonstrated in placebo-controlled studies in patients with moderate to severe AD [6]. Dupilumab showed significant clinical effects in the evaluation of disease severity, as measured by the Eczema Area and Severity Index (EASI), Investigator’s Global Assessment (IGA), and SCORing Atopic Dermatitis (SCORAD). In addition, treatment with dupilumab resulted in a significant reduction in itching. Dupilumab has been shown to be effective in both intrinsic and extrinsic AD [7]. Dupilumab is also approved for the treatment of prurigo nodularis, moderate to severe asthma, eosinophilic esophagitis, and chronic rhinosinusitis with nasal polyps and chronic spontaneous urticaria. This covers several Th2-associated inflammatory diseases. 

**Safety**

Treatment with dupilumab is generally well tolerated, so routine blood tests are not recommended [8]. However, a significant number of patients develop conjunctivitis (up to 20% depending on the study), which is mild to moderate in most cases [8, 9]. In clinical studies, the risk was 2.64 times higher compared to placebo, and in real-life studies, it averaged 13% [10]. In children, ocular symptoms have been reported in up to 35% of minors treated with dupilumab, although symptoms may not appear until more than a year after the start of therapy [11]. Topical treatment with eye drops, such as artificial tears, topical antihistamines, temporary topical steroids, or topical ciclosporin in treatment-resistant cases, is usually sufficient and does not require discontinuation of dupilumab. However, in severe cases of treatment-resistant conjunctivitis, consultation with an ophthalmologist is recommended [12]. For children up to preschool age with ocular symptoms, however, early ophthalmological care is recommended, as there is a risk that potential symptoms such as visual impairment or ocular pain are often not adequately identified [13]. 

In phase III trials, local reactions at the injection site were described more frequently than after placebo injections. However, these reactions are usually mild and have little clinical relevance. Nevertheless, injection pain and pain at the injection site can affect the acceptance of therapy, particularly in pediatric patients. 

Transient, clinically insignificant increases in eosinophils are not uncommon with dupilumab treatment. In adults treated with dupilumab for respiratory conditions, the clinical relevance of eosinophilia has rarely been reported (in seven out of 4,666 patients, six of whom had eosinophilic granulomatosis with polyangiitis) [14, 15]. Therefore, a cutoff value of 1,500 EOS/µL has been proposed for adults with respiratory symptoms to determine the need for therapy with this antibody [15, 16]. For adult patients with AD, especially those with additional respiratory diseases, it may be useful to determine blood eosinophil levels before starting therapy. If baseline values are greater than 1,500/µL, patients should be monitored for this in laboratory tests and clinically as part of follow-up examinations. 

In some patients with AD, the initial manifestation, exacerbation, or recurrence of diseases such as psoriasis vulgaris, rheumatoid arthritis, or Crohn’s disease has been observed during dupilumab therapy. These diseases involve IL-17-producing immune cells in their pathogenesis. Therefore, patients with these comorbidities undergoing dupilumab therapy should regularly report any recurrence or worsening of symptoms and undergo clinical examinations. Depending on the severity, switching to another systemic therapy may be necessary. 

**Monitoring**

According to the current European “Summary of Product Characteristics” (SmPC), no laboratory tests or instrumental examinations are required to monitor the therapy. However, the determination of eosinophils in the blood is particularly useful for adults with additional respiratory symptoms (see above). 

**Special considerations**

For patients with AD and prurigo nodularis, asthma, chronic obstructive pulmonary disease (COPD) with elevated eosinophil counts, allergic rhinoconjunctivitis with nasal polyps, eosinophilic esophagitis, chronic spontaneous urticaria, or bullous pemphigoid, dupilumab treatment may positively impact these conditions [17]. However, dupilumab has not yet been approved to treat the latter disease. 

### 4.2. Lebrikizumab [Table Table7]

**Mechanisms of action and efficacy**

Lebrikizumab is a highly specific humanized monoclonal antibody that binds to soluble interleukin 13 and selectively prevents the formation of the IL-13Rα1/IL-4Rα heterodimer receptor signaling complex. In two randomized, placebo-controlled, double-blind Phase III studies, children (12 years and older), adolescents, and adults with moderate to severe AD were randomized to receive placebo or subcutaneous injections of lebrikizumab (250 mg every two weeks, with an initial dose of 500 mg at the start of the study and again in week two) [18]. 

Complete or nearly complete healing of AD (IGA 0/1) was observed in 43% and 33% of patients in the lebrikizumab groups after 16 weeks, compared to 13% and 11% in the placebo group. The proportion of patients with a 75% improvement in the EASI score (EASI-75) was 59% and 52%, respectively, in the treatment groups, compared to 16% and 18%, respectively, in the placebo groups. Itching and sleep disturbances also improved significantly during therapy. Conjunctivitis was reported as an adverse effect. 

**Safety**

In the Phase III studies, treatment-related adverse events were observed in over 45% of patients in the treatment groups and over 51% of patients in the placebo groups. Most of these events were mild to moderate and did not lead to discontinuation of treatment. 

The most common adverse event occurring in at least 5% of patients receiving lebrikizumab, which was reported more frequently than in the placebo group, was conjunctivitis (7.4% vs. 2.8%, and 7.5% vs. 2.1% in the two Phase III studies, respectively) [18]. 

**Monitoring**

According to the current European SmPC, no laboratory tests or instrumental examinations are required to monitor the therapy. 

### 4.3. Nemolizumab[Table Table10]

**Mechanisms of action and efficacy**

Nemolizumab, a humanized monoclonal antibody, binds to the alpha chain of the interleukin-31 receptor (IL-31RA). It was originally developed to treat AD-related pruritus. 

A total of 1,728 adults and adolescents (ages 12 and older) with moderate to severe AD and associated pruritus who had previously responded inadequately to topical glucocorticoid therapy were enrolled in two randomized, double-blind, placebo-controlled Phase 3 studies (ARCADIA 1 and 2). Patients received 30 mg of nemolizumab or placebo injections every four weeks, in addition to TCS and, in some cases, topical calcineurin inhibitors (TCI). After 16 weeks, 44% (270/620) and 42% (220/522) of nemolizumab-treated patients achieved an EASI-75 response, compared with 29% (93/321) and 30% (80/265) of placebo-treated patients. The proportion of patients with an IGA response (defined as an IGA score of 0 or 1 with at least a 2-point improvement) was also significantly higher in the nemolizumab groups than in the placebo group. Secondary endpoints, such as reductions in pruritus and sleep disturbances, also showed significant benefits for nemolizumab [19]. 

After 16 weeks, patients who had a clinical response (IGA 0/1 or EASI-75) were randomized again (1 : 1 : 1) and received nemolizumab 30 mg Q4W, nemolizumab 30 mg Q8W, or a placebo for an additional 32 weeks. Additional topical anti-inflammatory therapy was permitted during this phase of the study. By week 48, a higher percentage of patients who continued nemolizumab treatment showed a sustained IGA 0/1 response (Q4W: 61.5%; Q8W: 60.4%) compared to the placebo group (49.7%). Accordingly, sustained EASI-75 response rates were higher in the nemolizumab groups (Q4W: 76.3%; Q8W: 75.7%) than in the placebo group (63.9%) [20]. 

**Safety**

In the two Phase III studies mentioned above, the incidence of adverse events was similar for nemolizumab and placebo. The most common treatment-related event was exacerbation of AD, which occurred in 12% (75/616) of patients in ARCADIA 1 and 11% (34/321) of patients on placebo. In ARCADIA 2, it occurred in 7% (37/519) of patients and 6% (15/263) of patients on placebo [19]. 

Both studies observed asthma attacks in both groups, although it should be noted that over 30% of the patients included in the studies had a history of asthma [19]. Patients with prurigo nodularis who received nemolizumab as part of a study also reported worsening asthma symptoms after starting treatment. This occurred more frequently in patients weighing more than 90 kg who received 60 mg of nemolizumab every four weeks (a dosage not approved for AD) than in patients weighing less than 90 kg who received 30 mg every four weeks [21]. 

Urticaria was reported in 1% to 2% of patients treated with nemolizumab in the AD-studies. None of these cases were classified as severe. By contrast, urticaria was observed in fewer than 1% of participants in the placebo groups [19]. 

**Monitoring**

According to the current European SmPC, no laboratory tests or instrumental examinations are required to monitor the therapy. 

**Special considerations**

For patients with AD and prurigo nodularis, nemolizumab treatment can also have positive effects on the condition [22]. 

### 4.4. Tralokinumab[Table Table8]

**Mechanisms of action and efficacy**

Tralokinumab, a fully human IgG4 (mAb) that neutralizes IL-13, was approved by the European Medicines Agency (EMA) in the summer of 2021. In two 52-week, double-blind, placebo-controlled Phase III studies, adults with moderate to severe AD were randomized to receive either 300 mg of subcutaneous tralokinumab or a placebo every two weeks [23]. After 16 weeks of treatment, tralokinumab monotherapy was superior to placebo. The primary endpoints were an IGA score of 0 or 1 and an EASI score of 75 by week 16. Patients who achieved an IGA score of 0 or 1 and/or an EASI score of 75 at week 16 while receiving tralokinumab were randomized again and received either tralokinumab every two weeks (Q2W) or every four weeks, or a placebo for 36 weeks. Most patients who responded to tralokinumab by week 16 maintained their response by week 52 with continued treatment without the need for rescue medication. Tralokinumab also achieved a higher rate of treatment success compared to placebo for AD of the head and neck, which is often particularly difficult to treat [24]. 

Phase III studies also examined what happens when patients who respond well to tralokinumab for 16 weeks continue treatment as before, reduce the frequency of treatment, or discontinue treatment. 

After 16 weeks, patients who achieved an EASI-75 or IGA success were randomized again. They could either continue treatment every two weeks, transition to treatment every four weeks, or receive a placebo. After 52 weeks, more than 55% of patients who received treatment every two weeks still achieved an EASI score of 75, as did approximately 50% of patients who received treatment once a month. Over 51% of patients who continued treatment every two weeks maintained an IGA score of 0 or 1, compared to 39% and 45% of patients who switched to treatment once a month, respectively. 

**Safety**

In the two Phase III studies, adverse events occurred in 76.4% and 61.5% of patients receiving tralokinumab and in 77.0% and 66.0% of patients receiving placebo during the 16-week initial phase, respectively. 

Ocular complications occurred less frequently with tralokinumab than with dupilumab [23]; a meta-analysis of four published studies reported an incidence rate of 6.2% in tralokinumab patients, compared to 2.1% for placebo [25]. 

**Monitoring**

According to the current European SmPC, no laboratory tests or instrumental examinations are required to monitor the therapy. 

## 5. Interval or long-term therapy with JAK inhibitors[Table Table5]

The Janus kinase (JAK) family, which includes JAK1, JAK2, JAK3, and tyrosine kinase 2 (TYK2), is a class of cytoplasmic tyrosine kinases [26]. They dock onto the intracellular part of various cytokine receptor chains, forming functional signaling complexes. These complexes regulate the inflammatory process by activating cytoplasmic transcription factors known as signal transducers and activators of transcription (STATs). When STAT proteins are activated, they form dimers that migrate to the cell nucleus to either positively or negatively regulate the expression of target genes for inflammatory mediators. Therefore, inhibition of JAK activity may be more effective than targeted inhibition of a single cytokine signaling pathway. However, since various approved JAK inhibitors affect the four JAKs to different degrees, their effects vary greatly. In addition to interrupting cutaneous proinflammatory cytokine signaling, JAK inhibition has been reported to rapidly alleviate chronic itching and improve skin barrier function by increasing the expression of filaggrin, a protein that plays a key role in skin barrier function [27, 28]. 

The broader mechanism of action compared to Th2-directed agents also explains the broader spectrum of possible adverse effects. For instance, the antiviral effect of type I interferons is inhibited, leading to an increased incidence of herpes simplex and herpes zoster infections, as well as an increased susceptibility to infection, particularly among older individuals. 

In autumn 2022, the EMA reviewed all available safety data for all approved JAK inhibitors. The indication for these inhibitors for the treatment of AD or alopecia areata remained unchanged. On March 10, 2023, the European Commission published its decision, completing the risk assessment procedure pursuant to Article 20 of Regulation (EC) No. 726/2004 on Janus kinase inhibitors. The warnings and precautions for use have been updated. According to this update and Direct Healthcare Profession Communication (Rote Hand Brief) dated March 17, 2023 [29], these medications should only be used in the following patients if no suitable treatment alternatives are available: Patients aged 65 years or older, patients with cardiovascular risk (e.g., heart attack or stroke), patients who smoke or are former long-term smokers, and patients with an increased risk of cancer. JAK inhibitors should be used with caution in patients with risk factors for blood clots in the lungs and deep veins (venous thromboembolism, VTE) who do not belong to the patient groups mentioned above. In addition, the dosage should be reduced whenever possible in patient groups at risk for venous thromboembolism, cancer, or severe cardiovascular problems. Regular skin examinations are also recommended for all patients [30]. 

The most suitable substance should be selected on a case-by-case basis with patient or parental involvement (i.e., shared decision-making). 

The German Society for Rheumatology (DGfR) recommends the following screening and ongoing monitoring before using JAK inhibitors for rheumatological indications [31]: 

**Examination program before starting therapy with JAK inhibitors**

- General status to rule out active infection
- Review and, if necessary, update vaccination status
- Hepatitis B screening
- Pregnancy test
- Testing for active or latent tuberculosis: Chest X-ray (not older than three months) and appropriate screening tests (preferably IGRA). If there are signs of latent TB, prophylaxis should be administered, if possible, four weeks before the start of therapy. This can be done with either isoniazid for nine months or rifampicin for four months. Strict indications and regular monitoring are required. This procedure is in accordance with the current recommendations of the German Society for Rheumatology (DGRh). However, the recently published S3 guideline “Therapy of Psoriasis vulgaris” does not generally recommend a chest X-ray anymore: https://register.awmf.org/assets/guidelines/013-001l_S3_Therapie-Psoriasis-vulgaris_2026-02.pdf [32].
- Laboratory tests: ESR, CRP, differential blood count, GOT, GPT, and creatinine. Lipid profile (total cholesterol, LDL, HDL, triglycerides).

For patients with an absolute lymphocyte count below 500/µL, an absolute neutrophil count below 1,000/µL, or a hemoglobin level below 8 g/dL, therapy with JAK inhibitors should either not be started or be temporarily paused. 

In clinical trials, creatine phosphokinase (CPK) levels often increased at the start of treatment with JAK inhibitors and remained stable at higher levels thereafter, even during long-term therapy. Cases of rhabdomyolysis are not mentioned in the current European SmPC in the EU for abrocitinib, baricitinib, or upadacinitib (as of 2025 and 2026, respectively). CPK monitoring is not mentioned in the rheumatological recommendations for monitoring JAK inhibitors baricitinib and upadacitinib due to the lack of clinical relevance. However, it would be recommended for high-performance athletes under JAK inhibition, for example. 

**Examination program during therapy with JAK inhibitors**

**Clinical examination**

- Signs of infection, particularly upper respiratory tract infections (cough), herpes zoster, fever, diarrhea, and unexplained weight loss

**Laboratory tests**

- Safety and activity parameters (ESR and/or CRP, differential blood count, GOT, and GPT) should be checked approximately every four weeks during the first three months. If values are stable and normal, they should be checked every eight to 12 weeks. Lipid values should be checked four to eight weeks after the start of therapy, then every six months.

Attention must be paid to any additional checks that may be necessary due to concomitant medication. 

**Zoster vaccination during oral therapy for AD with JAK inhibitors**

The incidence of herpes zoster generally increases during treatment with oral JAK inhibitors. A meta-analysis of clinical studies on upadacitinib for AD revealed a dose-dependent incidence of approximately three and five patients per 100 patient-years of treatment for 15 and 30 mg of upadacitinib per day, respectively [33]. 

As of November 6, 2025, the Standing Committee on Vaccination (STIKO) at the German Robert Koch Institute has expanded its recommendation for zoster vaccination. According to the new recommendation, all patients aged 18 and older taking immunosuppressive medications should be vaccinated against herpes zoster. JAK inhibitors are explicitly mentioned (https://www.rki.de/SharedDocs/FAQs/DE/Herpes_zoster/FAQ-Liste_Impfempfehlung.html) [34]. Patients who are already being treated with oral JAK inhibitors should also be vaccinated. On February 13, 2026, the eligibility requirements for preventive vaccinations against herpes zoster (shingles) were adjusted accordingly in Germany [35]. 

In a position paper published in 2019, the STIKO made a recommendation on how to proceed in this regard. This recommendation is analogous to the pan-JAK inhibitor tofacitinib [36]. Accordingly, vaccination should be completed at least two weeks, and preferably four weeks, before the start of therapy. If therapy has already begun, it is advisable to pause the JAK inhibitor for at least two to three days before vaccination until at least one week after vaccination, in accordance with the current recommendations for inactivated vaccines. 

## 5.1 Abrocitinib[Table Table11]

**Mechanisms of action and efficacy**

Like all JAK inhibitors, abrocitinib is a fast-acting drug. It is a selective, oral JAK1 inhibitor that has been shown to be effective in patients with moderate to severe AD as monotherapy (MONO-1 and MONO-2 studies) [37, 38] and in combination with topical therapies, compared to placebo, in terms of treatment response, as measured by IGA and EASI-75 scores (COMPARE study) [39]. In the MONO studies, the proportion of patients with an EASI-75 response at week 12 was significantly higher with abrocitinib 100 mg (~ 40 – 45%) and abrocitinib 200 mg (~ 61 – 63%) than with placebo (~ 10 – 12%) [37, 38]. In the COMPARE study, the proportion of patients with EASI-75 scores was also significantly higher with 100 mg and 200 mg of abrocitinib (~ 59% and ~ 70%, respectively) than with placebo (27%) [39]. Similar efficacy was demonstrated in the JADE TEEN study in adolescents when both the 100 mg and 200 mg doses were used in combination with topical therapy [40]. In the COMPARE study, higher response rates were observed with 200 mg of abrocitinib than with dupilumab in the subgroup with severe disease after 16 weeks of treatment. The efficacy of abrocitinib 100 mg and dupilumab was similar in this subgroup. The JADE-DARE study examined the efficacy and safety of abrocitinib 200 mg and dupilumab 300 mg in 727 adult patients. Abrocitinib was significantly more effective within the 2- to 8-week timeframe. The results suggest that patients with severe AD are more likely to respond to treatment with abrocitinib 200 mg than with dupilumab within this time frame [41]. 

**Safety**

A total of 2,856 patients participated in long-term follow-up studies, including Phase II and III studies and a long-term extension study. These studies involved 1,614 patient-years of exposure, with 1,248 patients exposed for ≥ 24 weeks and 606 patients exposed for ≥ 48 weeks (maximum 108 weeks). The following data were obtained: In the placebo-controlled cohort (n = 1,540), dose-dependent adverse events such as nausea (14.6%, 6.1%, and 2.0% for 200 mg, 100 mg, and placebo, respectively), headache (7.8%, 5.9%, and 3.5%), and acneiform exanthema (4.7%, 1.6%, and 0%) were observed. A transient, dose-dependent decrease in platelet count was observed: 2/2.718 in the 200 mg group had a confirmed platelet count of < 50 × 10³/mm³ at week 4. Incidence rates (IRs) were 2.33 and 2.65 per 100 person-years for severe infections, 4.34 and 2.04 per 100 person-years for herpes zoster, and 11.83 and 8.73 per 100 patient years for herpes simplex in the 200 mg and 100 mg groups, respectively [42]. 

Although clinical trials with abrocitinib did not reveal significant increases, caution should be exercised when using the substance in patients at increased risk of deep vein thrombosis or pulmonary embolism due to a potential class-wide effect associated with other JAK inhibitors, such as tofacitinib (see introduction to the substance class). 

**Special considerations**

Abrocitinib has not yet been tested in published controlled clinical studies for other inflammatory diseases. 

## 5.2 Baricitinib[Table Table12]

**Mechanisms of action and efficacy**

Like all JAK inhibitors, baricitinib is a fast-acting drug. It is an oral, selective inhibitor of JAK1 and JAK2. Its active ingredient was investigated in phase 2 and several phase 3 studies in adults with moderate to severe AD at doses of 1 mg, 2 mg, and 4 mg once daily compared to placebo. These studies showed significant improvements in EASI scores from baseline to 16 weeks, particularly at the two higher doses (2 and 4 mg daily) [43]. These studies demonstrated comparable efficacy in terms of the IGA score. Theoretically, baricitinib’s lower JAK selectivity as a JAK1/JAK2 inhibitor compared to abrocitinib and upadacitinib may have advantageous effects, such as greater efficacy, as well as disadvantageous effects, such as a broader spectrum of side effects, including those affecting JAK2-dependent hematopoiesis. However, the available data on the safety profile from clinical studies does not allow to draw this conclusion. One study permitted the simultaneous use of topical corticosteroids [44]. 

In October 2023, the European Medicines Agency (EMA) approved baricitinib for the treatment of children aged 2 years and older with moderate to severe AD. The approval was based on the results of the Phase 3 BREEZE-AD PEDS study, in which 483 pediatric patients (average age 12 years; ages 2 to 18 years) received baricitinib at doses of 0.5 mg, 1 mg, 2 mg, 4 mg, or a placebo for 16 weeks. The 4 mg dose of baricitinib (2 mg for patients under 10 years of age and 4 mg for patients 10 years of age and older) was superior to the placebo in achieving an Investigator Global Assessment response (score of 0 or 1 with an improvement of at least 2 points) as well as the secondary endpoints: EASI-75, EASI-90, mean reduction in EASI from baseline, SCORAD-75, and reduction in itching by at least 4 points on the numerical rating scale (NRS) [45]. 

**Safety**

The most common side effects observed in clinical trials of baricitinib include increased LDL cholesterol, upper respiratory tract infections, acneiform exanthema, and headaches. Herpes simplex has been reported among other infections associated with baricitinib. The overall incidence of these events was low, as identified in a combined safety study of 2,531 patients from eight RCTs who received baricitinib over 2,247 patient-years (median duration 310 days): eczema herpeticum (n = 11), erysipelas (n = 6), and pneumonia (n = 3). Patients with a history of recurrent eczema herpeticum in the previous year were excluded from baricitinib clinical trials, as well as from trials with abrocitinib and upadacitinib. Four opportunistic infections were reported [46]. Short-term increases in CPK are possible, particularly following intense physical exertion. During the placebo-controlled period, no cases of malignant disease, gastrointestinal perforation, confirmed cardiovascular event, or tuberculosis were reported in patients treated with baricitinib. The incidence of herpes simplex infection was higher in the 4 mg group (6.1%) than in the 2 mg group (3.6%) or the placebo group (2.7%). Currently, long-term safety data beyond 16 weeks is not available for AD. 

Although clinical trials with baricitinib did not demonstrate significant increases, caution should be exercised when using it in patients at increased risk of deep vein thrombosis or pulmonary embolism due to a potential class effect with other JAK inhibitors, such as tofacitinib (see introduction to the substance class). 

In the pediatric BREEZE-AD PEDS study, the most common adverse events were abdominal pain, acneiform rash, and headache. Only a few patients discontinued treatment due to adverse events: 1.6% in the placebo group and 0.6% in the baricitinib group [45]. 

**Special considerations**

Patients with AD who suffer from inflammatory comorbidities such as rheumatoid arthritis, juvenile idiopathic arthritis, or alopecia areata are likely to experience positive effects. Baricitinib is already approved for these indications. 

## 5.3 Upadacitinib[Table Table13]

**Mechanisms of action and efficacy**

Like all JAK inhibitors, upadacitinib is a fast-acting drug. It is another JAK 1 inhibitor. A phase 2 study involving 167 adult patients investigated three different doses of upadacitinib (30 mg/day, 15 mg/day, and 7.5 mg/day) for the treatment of AD in comparison with a placebo [47]. The study ran for 16 weeks. In all dosage groups, upadacitinib was superior to placebo in terms of EASI (mean change (SE): 74% (6.1%) for 30 mg, 62% (6.1%) for 15 mg, 39% (6.2%) for 7.5 mg, and 23% (6.4%) for placebo (p = 0.03, < 0.001, < 0.001)). Significant improvements were also observed in terms of the SCORAD index, the NRS scale for pruritus, and the POEM scale. Studies published since then have shown similar efficacy [48, 49]. In a comparative study of 30 mg of upadacitinib versus dupilumab, 247 patients (71.0%) receiving upadacitinib and 210 patients (61.1%) receiving dupilumab achieved EASI-75 (p = 0.006) [50]. Upadacitinib demonstrated superiority over dupilumab in several secondary endpoints. These included an improvement in worst pruritus NRS as early as week 1 (mean (SE), 31.4% (1.7%) vs. 8.8% (1.8%); p < 0.001), the achievement of EASI-75 by week 2 (152 (43.7%) vs. 60 (17.4%); p < 0.001), and the achievement of EASI-100 by week 16 (97 (27.9%) vs. 26 (7.6%); p < 0.001). The superiority of 30 mg of upadacitinib was particularly evident at the beginning of the treatment period. However, severe infections, eczema herpeticum, herpes zoster, and laboratory-related adverse events occurred more frequently with upadacitinib, while conjunctivitis occurred more frequently in patients treated with dupilumab. 

**Safety**

The cumulative incidence rates of adverse events in the Phase II study were 78.6% for the 30 mg group, 76.2% for the 15 mg group, 73.8% for the 7.5 mg group, and 61% for the placebo group. These rates have remained consistent in subsequent studies [47]. The most common adverse effects of upadacitinib were upper respiratory tract infections and acneiform exanthema. Herpes zoster infections occurred at a frequency that was dose-dependent and roughly comparable to that observed for other indications of upadacitinib [33]. The risk of eczema herpeticum was only slightly increased; however, patients with recurrent episodes or an episode within the past 12 months were excluded from inclusion in the clinical trials (which also applies to studies with the other JAK inhibitors baricitinib and abrocitinib) [33]. 

Other side effects, such as nausea and headaches, were particularly pronounced at the start of therapy. Cumulative incidence rates of serious adverse events were 0% for the 30 mg group, 2.4% for the 15 mg group, 4.8% for the 7.5 mg group, and 2.4% for the placebo group. There were no reported discontinuation rates [47]. 

Although clinical trials with upadacitinib did not reveal significant increases, caution should be exercised when using the substance in patients at increased risk of deep vein thrombosis or pulmonary embolism due to a potential class-wide effect associated with other JAK inhibitors, such as tofacitinib (see introduction to the substance class). 

**Special considerations**

AD patients who suffer from inflammatory comorbidities, such as rheumatoid arthritis, psoriatic arthritis, ankylosing spondylitis, axial spondyloarthritis, ulcerative colitis, Crohn’s disease, or giant cell arteritis, are likely to experience positive effects. Upadacitinib is already approved for these conditions. 

## 6. Systemic therapy during pregnancy and breastfeeding 

### 6.1 Pregnancy [Table Table14]

The long-term use of systemic glucocorticosteroids for AD is generally not recommended, especially during pregnancy. This is because it is associated with an increased risk of fetal complications, including gestational diabetes [51]. Only the short-term administration of prednisolone (up to 0.5 mg/kg/day) is permitted under strict conditions. 

Ciclosporin may be used during pregnancy for severe, uncontrolled AD when topical anti-inflammatory therapy alone or in combination with ultraviolet (UV) treatment fails and better long-term disease control is necessary. However, particular attention should be given to monitoring the mother’s kidney function and blood pressure. There is no evidence of teratogenicity. Ciclosporin crosses the placenta [52] and should therefore only be used during pregnancy if the potential benefit to the mother outweighs the potential risk to the fetus. Close consultation with an experienced gynecologist is strongly recommended when prescribing this drug. 

Off-label use of azathioprine may be considered for severe, uncontrolled AD during pregnancy if the patient was already receiving azathioprine treatment at the time of conception. Studies of patients with inflammatory bowel disease have found no evidence of teratogenicity. Nevertheless, close consultation with an experienced gynecologist is strongly recommended when prescribing the drug [53]. 

Methotrexate and mycophenolate mofetil are expressly contraindicated during pregnancy because they are teratogenic. 

Currently, there is no clinical data available on the new systemic therapies that provides information about possible drug-related risks. Although preclinical data do not suggest that dupilumab, lebrikizumab, nemolizumab, or tralokinumab are teratogenic when used during pregnancy, their use is not recommended due to a lack of experience with their use in pregnant patients. 

Due to its earlier approval and existing evidence base, dupilumab is being evaluated separately from tralokinumab, lebrikizumab, and nemolizumab. According to the European SmPC, dupilumab may be used during pregnancy under special circumstances. The European SmPC states, “Dupilumab should only be used during pregnancy if the potential benefit justifies the potential risk to the fetus” [17]. Current clinical data does not indicate an association between dupilumab treatment and negative pregnancy outcomes [54, 55, 56, 57, 58, 59, 60, 61]. 

However, dupilumab antagonizes the Th2 physiological environment during pregnancy, which tolerates fetal alloantigen’s. This could pose an immunological risk. 

Retrospective clinical data from an Italian multicenter study comparing 29 pregnant women exposed to dupilumab with a control group is available. With an average exposure duration of six weeks (range: two to 24 weeks), no statistically significant negative effects were observed [62]. A group of experts in the field of allergology concluded in 2025 based on published data that a therapy with dupilumab is probably not associated with an increased risk in the first trimester [63]. 

Data from the TriNetX Registry were used to retrospectively examine the risk of maternal pregnancy complications in 293 women with type 2 inflammatory diseases (T2ID) who were undergoing dupilumab treatment. Using propensity score matching, the study compared women with and without dupilumab exposure in terms of demographics, diagnoses, medications, and risk factors for pregnancy complications. The results indicate that women treated with dupilumab during pregnancy did not have an increased risk of adverse outcomes. From the guideline authors’ perspective, the evaluation provides valuable new information on over 250 pregnancies treated with dupilumab. However, women who had previously reported a complication under investigation (e.g., miscarriage) were excluded from the evaluation, which introduces a moderate risk of bias (i.e., underestimation of an existing risk) [60]. 

Furthermore, conflicting results from evaluations of the VigiBase pharmacovigilance database regarding the use of dupilumab during pregnancy have not been included in the current assessment of the data. Limitations of VigiBase analyses and interpretations include data heterogeneity, lack of a control population, and lack of information on gestational age at the time of dupilumab therapy and atopic disease severity [61, 64, 65]. 

Initially, there was no clear consensus within the guideline committee regarding the use of dupilumab during pregnancy. However, the above recommendation that dupilumab should not be used during pregnancy due to a lack of experience was ultimately agreed upon unanimously. 

Based on their approval status, abrocitinib, baricitinib, and upadacitinib are contraindicated during pregnancy. There is no clinical data supporting their safety; only individual case reports exist. However, teratogenic effects have been observed in animal models for these active substances. 

Antihistamines are only moderately effective against AD-associated itchiness, yet they are frequently used in routine practice. If necessary, loratadine should be used as the drug of choice due to extensive experience with its use in pregnant women. 

### 6.2 Breastfeeding [Table Table6]

Short-term treatment with glucocorticosteroids is safe during breastfeeding, as only 0.1% of the dose taken by the mother is excreted in breast milk. 

Methotrexate, Azathioprine and JAK inhibitors are excreted in breast milk and may cause immunosuppression in newborns. Therefore, these medications are generally not recommended for breastfeeding mothers [53]. 

According to the current European SmPC, methotrexate therapy is contraindicated during breastfeeding, as the drug can pass into breast milk and potentially cause toxic effects in the breastfed infant [66]. However, only low concentrations of methotrexate are detected in breast milk, there is no evidence that this harms breastfed infants. The European Alliance of Associations for Rheumatology (EULAR) therefore recommends considering a “low-dose” therapy regimen if no other treatment can be used during breastfeeding [67]. According to the Embryotox database, however, it is recommended to wean the child or switch to a different therapy during breastfeeding. The database also notes that some authors recommend suspending breastfeeding for several half-lives. According to Embryotox, however, some authors consider breastfeeding during low-dose therapy to be acceptable, as harm to the infant appears unlikely due to the low transfer of the active ingredient into breast milk [68]. 

Regarding azathioprine, the European SmPC states that 6-mercaptopurine was de tected in the colostrum and breast milk of mothers treated with azathioprine [69]. Due to limited data availability, it is impossible to confirm or refute any risks to newborns or infants associated with azathioprine. The Embryotox database does not advise against azathioprine therapy during breastfeeding. It recommends checking the child’s/infant’s blood count in case of suspicion, as well as consulting the pediatrician about the mother’s medication [70]. Due to the very low concentration of ciclosporin in breast milk and experience in transplant medicine, the use of ciclospo rin is not contraindicated in breastfeeding women [71, 72]. A 2023 Delphi consensus by a Northern European interdisciplinary group of experts recommended ciclosporin as the preferred long-term (first-line) therapy during breastfeeding [73]. Nevertheless, the Eu ropean SmPC recommends against breast feeding during ciclosporin treatment due to possible serious side effects in breastfed newborns and infants [74]. According to Embryotox, mothers should inform their pe diatrician if they are undergoing ciclosporin therapy while breastfeeding [75]. There is currently insufficient clinical data available to make a recommendation for the use of the biologics dupilumab, leb rikizumab, nemolizumab, and tralokinumab in breastfeeding mothers. The aforementioned Delphi consensus of an interdisciplinary group of Northern European experts also stated that women with AD who wish to breastfeed should be encouraged to do so. If a sufficient response is not produced by topical therapy with TCS or TCI, it is recommended that breastfeeding patients should be fully informed about the potential risks and contraindications of sys temic therapies as part of a shared decision making process [73]. 

## Funding 

This guideline received funding by the German Dermatological Society (DDG). No influence on the guideline content occurred. 

## Conflict of interest 

The information on conflicts of interests (COI) was collected using the AWMF form of 2026 and assessed by the Division of Evidence-Based Medicine (dEBM Charité Berlin) for a thematic relation to the guideline. Low COI were depicted as sponsored lectures or authorship with a maximum of 1.500€ per year. Moderate COI were depicted as sponsored advisory or review activities (irrespective of the honoraria) or as sponsored lectures or as authorship exceeding 1.500€ per year. High COI were depicted as share ownerships irrespective of the amount. For final approval of the recommendations, only the votes of experts with no or only low conflicts of interest were taken into account, except for the chapter “Therapeutic Goals, Treatment Expectations, and Adjustment Criteria (‘Treat-to-Target’) in Systemic Therapies for AD” on which all delegates were allowed to vote. 

A full list of declared interests is available in the guideline development report (Leitlinienreport) at https://register.awmf.org/de/leitlinien/detail/013-027.

## Lead medical societies:

- DDG – German Dermatological Society e.V.

Further medical societies and organizations involved:

- Working Group Health Economics and Evidence-Based Medicine of the DDG
- Working Group Occupational and Environmental Dermatology of the DDG
- Working Group Pediatric Dermatology of the DDG
- Working Group Psychosomatic Dermatology of the DDG
- Dermatology Section of the DGAKI / Working Group Allergology of the DDG
- German Contact Allergy Group of the DDG
- BVDD – Professional Association of German Dermatologists e.V.
- ÖGDV – Austrian Society of Dermatology and Venereology
- SGDV – Swiss Society of Dermatology and Venereology
- Working Group Allergology of the SGDV
- AGNES – Working Group for Atopic Dermatitis Education e.V.
- DAAB – German Allergy and Asthma Association e.V.
- BVKJ – Professional Association of Pediatricians and Adolescent Medicine e.V.
- GPA – Society for Pediatric Allergology and Environmental Medicine e.V.
- DGKJ – German Society for Pediatric and Adolescent Medicine e.V.
- DGPM – German Society for Psychosomatic Medicine and Medical Psychotherapy e.V.
- DNVF – German Network for Health Services Research e.V.
- DNP – German Atopic Dermatitis Association e.V.
- DGpRP – German Society for Pediatric Rehabilitation and Prevention e.V.
- DGAKI – German Society for Allergology and Clinical Immunology e.V.
- Working Group Food Allergy of the DGAKI
- Pediatrics Section of the DGAKI
- DEGAM – German Society for General Medicine and Family Medicine
- European Task Force for Atopic Dermatitis
- VDOE – Professional Association for Oecotrophology

**Table Table4:** 

**Symbol**	**Strength of recommendations**
**↑↑**	Strong recommendation: shall
**↑**	Recommendation: should
**0**	Recommendation may be considered
**↓**	Recommendation: should not
**↓↓**	Strong recommendation: shall not
	No recommendation

Adapted from the AWMF recommendation https://www.awmf.org/regelwerk/formulierung-und-graduierung-von-empfehlungen.

**Table Table9:** Treatment goals and adjustment criteria for systemic therapy in atopic dermatitis

The treatment goals, expectations, and adjustment criteria **shall** be established through a shared decision-making process with patients at the start of systemic therapy and reviewed regularly.	**↑↑**	100% consensus-based
		
A change in systemic therapy **shall** be discussed with patients if an EASI-50 has not been achieved 3 – 4 months after the initiation of systemic therapy.	**↑↑**	> 75% consensus-based
A change in systemic therapy **should** be discussed with patients if, 3 to 4 months after the initiation of systemic therapy, one or more of the following criteria is not met: EASI-75, a ≥ 4-point improvement in the DLQI, a ≥ 3-point improvement in the itch NRS.	**↑**	
A change in systemic therapy **shall** be discussed with patients if, 6 months after the initiation of systemic therapy, at least two of the following criteria have not been met: EASI-75, EASI score < 7, regardless of the baseline value at the start of systemic therapy Itching NRS ≤ 3 points, regardless of the baseline value at the start of systemic therapy, DLQI ≤ 5 points, regardless of the baseline value at the start of therapy.	**↑↑**	> 95% consensus-based
		
A change in systemic therapy** should **be discussed with the patients if an EASI-90 has not been achieved 6 months after the initiation of systemic therapy.	**↑**	> 75% consensus-based

**Table Table3:** 

Dupilumab **shall** be used in patients aged 12 years and older with moderate to severe AD, as well as in patients aged 6 months to 11 years with severe AD who are candidates for systemic therapy.	**↑↑**	100% evidence-based and consensus-based
		
Dupilumab **shall** be used as long-term therapy in AD.	**↑↑**	100% consensus-based
		
Dupilumab **shall** be used in patients with AD who are eligible for systemic therapy, particularly if they have concurrent bronchial asthma or concurrent chronic obstructive pulmonary disease with elevated blood eosinophil counts, or concurrent chronic rhinosinusitis with nasal polyps, or concurrent prurigo nodularis, or concurrent eosinophilic esophagitis, or concurrent chronic spontaneous urticaria, or concurrent bullous pemphigoid.	**↑↑**	100% consensus-based

**Table 1.** General recommendations for long-term systemic anti-inflammatory therapy in adult patients with AD who are eligible for systemic therapy (for details, see corresponding chapter).

	**Conventional systemic treatments**				**Biologics**				**JAK inhibitors**		
	**Azathioprine**	**Ciclosporin**	**Methotrexate**	**Mycofenolate mofetil**	**Dupilumab**	**Lebrikizumab**	**Nemolizumab**	**Tralokinumab**	**Abrocitinib**	**Baricitinib**	**Upadacitinib**
Recommendation	**0**	**0**	**↑**	**0**	**↑↑**	**↑↑**	**↑↑**	**↑↑**	**↑↑**	**↑↑**	**↑↑**
Dosage for adults^1^	Off-label; commonly used dosage adults: 1 – 3 mg/kg p.o. per day	licensed ≥ 16 years; commonly used dosage adults: 2.5; 5 mg/kg p.o. per day in two single doses	off-label; commonly used dosage adults: initial dose: 5 – 15 mg p.o. or s.c. per week; maximum dose: 25 mg/per week	off-label; commonly used dosage adults: 2 g p.o. per day in two single doses	licensed ≥ 6 months; Dosage for adults: initial 600 mg s.c. on day 1 followed by 300 mg every 2 weeks	licensed ≥ 12 years; Dosage for adults: 500 mg s.c. on days 1 and 15, followed by 250 mg every two weeks. If response is positive after 16 weeks, reduction of the frequency to every 28 days	licensed ≥ 12 years; Dosage for adults: 60 mg s.c. on day 1, followed by 30 mg every 4 weeks. If response is positive after 16 weeks, reduction of the frequency to every 8 weeks	licensed ≥ 12 years; dosage for adults: initial 600 mg s.c. on day 1 followed by 300 mg every 2 weeks; if response is positive after 16 weeks, reduction of the frequency to every 28 days	licensed ≥ 12 years; Dosage for adults: 100 or 200 mg p.o. per day; Age ≥ 65: 100 mg per day	licensed ≥ 2 years, Dosage for adults: 2 or 4 mg p.o. per day; Age ≥ 65: 2 mg per day	licensed ≥ 12 years; Dosage for adults: 15 or 30 mg p.o. per day; Age ≥ 65: 15 mg per day
Time to response (weeks)^2^	8 – 12	1 – 2	8 – 12	No data	4 – 6	4 – 6	4 – 8	6 – 8	1 – 2	1 – 2	1 – 2
Time to relapse (weeks)^2^	> 12	< 2	> 12	No data	> 8	> 8	> 8	> 8	< 2	< 2	< 2
Selection of most relevant adverse events	Gastrointestinal complaints, idiosyncratic hypersensitivity, hepatotoxicity, myelotoxicity	Serum creatinine ↑, blood pressure↑	Nausea, fatigue, transaminases ↑, myelotoxicity	Gastrointestinal complaints, headaches, myelotoxicity	Conjunctivitis, upper respiratory tract infections	Conjunctivitis, upper respiratory tract infections, eczema	Upper respiratory tract infections	Conjunctivitis, Upper respiratory tract infections	Upper respiratory tract infections, H. zoster, acneiform exanthema	Upper respiratory tract infections, H. zoster, acneiform exanthema	Upper respiratory tract infections, H. zoster, acneiform exanthema

^1^SmPC; ^2^expert experience, ↑ rise.

**Table Table5:** 

Treatment of AD with JAK inhibitors is approved for long-term therapy, but **should **also be used as intermittent therapy in certain clinical presentations (e.g., cases characterized primarily by seasonal flare-ups).	**↑**	100% consensus-based
		
Before using JAK inhibitors, screening **shall** be performed, and monitoring **shall** be carried out during treatment.	**↑↑**	100% consensus-based
		
The individual risk of serious infections **shall** be carefully assessed before using JAK inhibitors.	**↑↑**	100% consensus-based
		
When using systemic JAK inhibitors, zoster vaccination **shall **be administered starting at age 18, in accordance with the STIKO recommendation.	**↑↑**	100% consensus-based
		
JAK inhibitors **shall not** be used in patients with a history of thromboembolic events or a genetically determined increased risk of thrombosis.	**↓↓**	100% consensus-based

**Table Table6:** 

In breastfeeding women with AD, prednisolone **should only be considered** as a short-term rescue therapy for acute flare-ups.	**↑**	100% consensus-based
		
JAK inhibitors and mycophenolate mofetil **shall not** be used in breastfeeding women with AD.	**↓↓**	100% consensus-based

**Table Table7:** 

Lebrikizumab **shall** be used in patients aged 12 years and older with moderate to severe AD who are candidates for systemic therapy.	**↑↑**	100% evidence-based and consensus-based
		
If the patient responds well to the therapy, the interval between lebrikizumab injections **shall** be extended from two to four weeks. If the initial response is only partial, the interval between lebrikizumab injections **shall** be every two or four weeks until week 24, depending on the clinical severity of AD.	**↑↑**	100% consensus-based
If there is incomplete response after week 24, a switch to another systemic therapy or continuation of the bi-weekly therapy with lebrikizumab (off-label) **may be considered**.	**0**	
		
Lebrikizumab **shall** be used as a long-term therapy for AD.	**↑↑**	100% consensus-based

**Table Table8:** 

Tralokinumab **shall** be used in patients aged 12 years and older with moderate to severe AD who are candidates for systemic therapy.	** ↑↑**	100% evidence-based and consensus-based
		
Depending on the response to therapy and the clinical severity of AD, extending the injection interval for tralokinumab from two to four weeks **may be considered**.	**0**	100% consensus-based
		
Tralokinumab **shall** be used as a long-term therapy for AD.	**↑↑**	100% consensus-based

**Table 2.** General recommendations for long-term systemic anti-inflammatory therapy in children and adolescents and pregnant and breastfeeding patients with AD who are eligible for systemic therapy (for details, see corresponding chapter).

	**Conventional systemic treatments**				**Biologics**				**JAK inhibitors**		
	**Azathioprine**	**Ciclosporin**	**Methotrexate**	**Mycofeno-late mofetil**	**Dupilumab**	**Lebrikizumab**	**Nemolizumab**	**Tralokinumab**	**Abrocitinib**	**Baricitinib**	**Upadacitinib**
Children ≥ 6 months to < 2 years	**-**	**-**	**↑**	**0**	**↑↑**	**–**	**–**	**–**	**–**	**–**	**–**
Children ≥ 2 years to < 12 years										**↑**	
Adolescents ≥ 12 years to < 18 years						**↑↑**	**↑↑**	**↑↑**	**↑↑**	**↑↑**	**↑↑**
Dose for children/adolescents^1^	Off-label use with regard to indication; commonly used dosage for adults: 1 – 3 mg/kg p.o. per day	Licensed ≥ 16 years, commonly used dosage for children: 2.5 – 5 mg/kg p.o. per day in two separate doses; if the patient responds well, the lowest possible dosage should be aimed for	Off-label use with regard to indication; commonly used dosage for children: 0.3 – 0.4 mg/kg per week	Off-label use with regard to indication; commonly used dosage for children: 30 – 50 mg/kg per day	Licensed ≥ 6 months; dosage for children: Ages 6 months to 5 years From 5 kg to < 15 kg, initial dose of 200 mg s.c. on day 1 followed by 200 mg every 4 weeks. From 15 kg to < 30 kg, initial dose of 300 mg s.c. on day 1 followed by 300 mg every 4 weeks Age 6 – 11 years: from 15 kg < 60 kg, initial 300 mg s.c. on days 1 & 15 followed by 300 mg every 4 weeks for ≥ 60 kg, initial 600 mg s.c. on day 1 followed by 300 mg every 2 weeks age 12 – 17 years: < 60 kg: initial 400 mg s.c. on day 1 followed by 200 mg every 2 weeks For ≥ 60 kg: initial 600 mg s.c. on day 1 followed by 300 mg every 2 weeks	Licensed n ≥ 12 years; dosage for adolescents: 12 – 17 years, initial dose of 500 mg s.c. on days 1 and 15, followed by 250 mg every 2 weeks; if response is observed after 16 weeks, reduce frequency to every 4 weeks	Licensed ≥ 12 years; dosage for adolescents aged 12 – 17 years: initial dose of 60 mg s.c. on day 1, followed by 30 mg every 4 weeks; if response is observed after 16 weeks, reduce frequency to every 8 weeks	Licensed ≥ 12 years; dosage for adolescents: 12 – 17 years, initial dose of 600 mg s.c. on day 1, followed by 300 mg every 2 weeks; if response is observed after 16 weeks, reduce frequency to every 4 weeks	Licensed ≥ 12 years; dosage for adolescents: 12 – 17 years: 25 kg – < 59 kg 100 mg p.o. per day; ≥ 59 kg 100 or 200 mg per day	Licensed ≥ 2 years; dosage for children ≥ 2 years: 10 – < 30 kg: 2 mg p.o. per day; ≥ 30 kg: 4 mg per day; reduction to 2 mg per day possible depending on response to treatment	Licensed ≥ 12 years; dosage for adolescents: 12 – 17 years (≥ 30 kg body weight): 15 mg p.o. per day
Pregnancy	–	↑	↓↓	↓↓	↓	↓↓	↓↓	↓↓	↓↓	↓↓	↓↓
Breastfeeding	–	–	–	↓↓	–	–	–	–	↓↓	↓↓	↓↓

^1^SmPC.

**Table Table10:** 

Nemolizumab **shall **be used in patients aged 12 years and older with moderate to severe AD who are candidates for systemic therapy.	**↑↑**	100% evidence-based and consensus-based
		
Nemolizumab **shall** be used as a long-term therapy for AD.	**↑↑**	100% consensus-based
		
Nemolizumab **shall** be used particularly in patients with AD who are candidates for systemic therapy and who also have prurigo nodularis.	**↑↑**	100% consensus-based

**Table Table11:** 

Abrocitinib **shall** be used in patients aged 12 years and older with moderate to severe AD who are candidates for systemic therapy.	**↑↑**	100% evidence-based and consensus-based
Treatment with abrocitinib **shall** be initiated in patients aged 18 to 64 years with severe AD, after ruling out contraindications, at the higher dosage approved for this indication. Following a response to therapy, the dosage **shall** be adjusted according to clinical response.	**↑↑**	

**Table Table12:** 

Baricitinib **shall** be used in patients aged 12 years and older with moderate to severe AD who are candidates for systemic therapy.	** ↑↑**	100% evidence-based and consensus-based
Baricitinib **should** be used in children aged 2 to 11 years with moderate to severe AD who are candidates for systemic therapy.	**↑**	
		
Treatment with baricitinib **shall** be initiated in patients aged 18 to 64 years with severe AD, after ruling out any contraindications, at the higher dose approved for this indication.	** ↑↑**	100% consensus-based
After response to therapy, a dose reduction **may be considered **based on the individual benefit-risk assessment and the clinical course.	**0**	
		
Baricitinib **shall** be used particularly in patients with AD who are candidates for systemic therapy and who have concurrent alopecia areata or concurrent rheumatoid arthritis or juvenile idiopathic arthritis.	**↑↑**	100% consensus-based

**Table Table13:** 

Upadacitinib **shall** be used in patients aged 12 years and older with moderate to severe AD who are candidates for systemic therapy.	**↑↑**	100% evidence-based and consensus-based
		
Treatment with upadacitinib **shall** be initiated in patients aged 18 to 64 years with severe AD, after ruling out contraindications, at the higher dose approved for this indication, following a response to the therapy, the dosage should be adjusted based on clinical response.	**↑↑**	100% consensus-based
		
Upadacitinib **shall** be used particularly in patients with AD who are candidates for systemic therapy and who have concurrent rheumatoid arthritis, psoriatic arthritis, ankylosing spondylitis, ulcerative colitis, giant cell arteritis, or Crohn‘s disease.	**↑↑**	100% consensus-based

**Table Table14:** 

For pregnant women with AD who are candidates for systemic treatment, the use of ciclosporin **should **be considered (off-label).	**↑**	100% consensus-based
		
Pregnant women with AD **shall not** undergo long-term therapy with systemic glucocorticosteroids – this applies equally to all patients with AD.	**↓↓**	100% consensus-based
		
JAK inhibitors, methotrexate, and mycophenolate mofetil **shall not** be used during pregnancy.	**↓↓**	100% consensus-based
		
Dupilumab **should not** be used during pregnancy due to a lack of experience. If pregnancy is planned within the next 12 weeks, dupilumab **should not** be initiated.	**↓**	100% consensus-based
		
Lebrikizumab, nemolizumab, and tralokinumab **shall not** be used during pregnancy due to a lack of experience. If a patient is planning to become pregnant within the next 12 weeks, lebrikizumab, nemolizumab, and tralokinumab **shall not** be used.	**↓↓**	100% consensus-based
		
After discontinuing a biologic therapy for AD during pregnancy, it **should **be determined – based on the severity of the AD and after weighing the overall benefit-risk profile – whether topical therapy alone and/or UV therapy is sufficient, or whether there is an indication to switch systemic therapy to cyclosporine.	**↑**	100% consensus-based
